# 2685. Impact of Education and Clinic Site Champion on Extragenital Testing for Sexually Transmitted Infection

**DOI:** 10.1093/ofid/ofad500.2296

**Published:** 2023-11-27

**Authors:** Victoria Cunningham, Hayden L Smith, Jonathan Hurdelbrink, Katherine Sittig, Lisa A Veach

**Affiliations:** University of Iowa, Iowa City, Iowa; UnityPoint Health, Des Moines, Iowa; UnityPoint Health - Des Moines, Des Moines, Iowa; University of Maryland, Baltimore, Maryland; UnityPoint Health, Des Moines, Iowa

## Abstract

**Background:**

Over the last several years sexually transmitted infections (STIs) have been on the rise in the United States. A potential area for improving the diagnosis of STIs is appropriate testing of extragenital (EG) sites (i.e., throat or rectum). These sites may serve as a reservoir for infection and lead to increased transmission if not identified and treated. Prior studies have shown that in men who have sex with men, genital testing alone misses >70% of STIs. In other patient groups, this may range from 10-40%. The aims of this project were twofold: to determine the proportion of patients receiving EG STI testing, and to observe the effect of a two-phase educational intervention on EG site testing.

**Methods:**

Phase I (Jul-Dec 2021) included four urgent care clinics within a Midwest health system. Retrospective baseline data was ascertained for all adults receiving STI testing for Chlamydia trachomatis (CT) and Neisseria gonorrhea (GC) at these clinics. Next, all four clinics received access to an educational presentation regarding the importance of EG testing for STI detection. Two of these clinics served as pilot sites for the implementation of patient self-collection kits. Data was compiled for all adults undergoing STI testing at these clinics for a 6-month period. Phase II (Jul 2022-Mar 2023) involved further expansion and utilization of a site champion at several family medicine clinics including on-site promotion of multi-site testing and patient self-collection. Educational materials as in Phase I were provided to site champions

**Results:**

Phase I baseline data indicated that less than 1% of samples from patients receiving STI testing were obtained from EG sites. There was a small increase in EG site testing after the educational intervention and implementation of self-collected swab kits (Table 1). Phase II data showed an increase in EG testing after the utilization of a clinic site champion and the sharing of testing data in family medicine clinics (Table 2).

**Figure:**
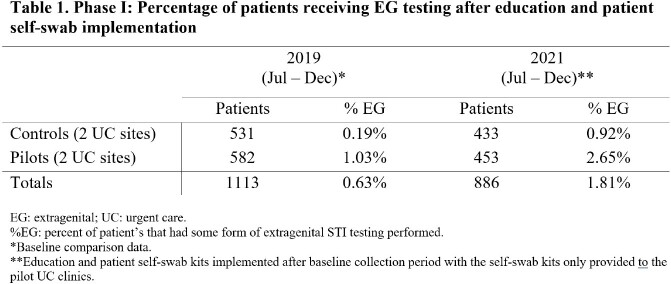

**Figure:**
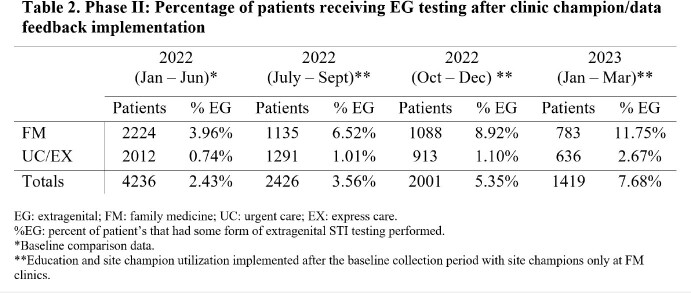

**Conclusion:**

Rates of STIs have reached an all-time high in the United States. EG infections serve as potential sources for STI transmission. Adequate multi-site testing is necessary. Implementation was most effective when a clinic site champion was identified, and clinic testing data was provided to them.

**Disclosures:**

**All Authors**: No reported disclosures

